# Protocol to target a promoter region in human embryonic kidney cells using the CRISPR-dCas9 system for single-locus proteomics

**DOI:** 10.1016/j.xpro.2024.103045

**Published:** 2024-04-30

**Authors:** Reem Alkhayer, Viviane Ponath, Elke Pogge von Strandmann

**Affiliations:** 1 Institute for Tumor Immunology, Philipps University Marburg, 35043 Marburg, Germany

**Keywords:** Genomics, ChIPseq, CRISPR

## Abstract

The unbiased identification of less-abundant transcription factors, which direct the expression of a target gene, is technically challenging. Here, we present a protocol to analyze the locus-specific chromatin-regulating proteome using *in situ* capture of chromatin interactions by an inactive Cas9 (dCas9). We describe steps for designing guide RNAs and transfection, followed by precipitation of chromatin and associated proteins. In the last step, we describe the elution of DNA and proteins for PCR and mass spectrometric analysis, respectively.

For complete details on the use and execution of this protocol, please refer to Alkhayer et al.^1^

## Highlights

• Locus-specific targeting using CRISPR-dCas9 system
• Steps to precipitate DNA and proteins using a Cas9 antibody
• Approach for single-locus proteomics of the targeted region
• Technique which may be applied generally to study promoter regulation

## Abstract (editor highlights)

Publisher’s note: Undertaking any experimental protocol requires adherence to local institutional guidelines for laboratory safety and ethics.

## Graphical abstract

**Figure:**
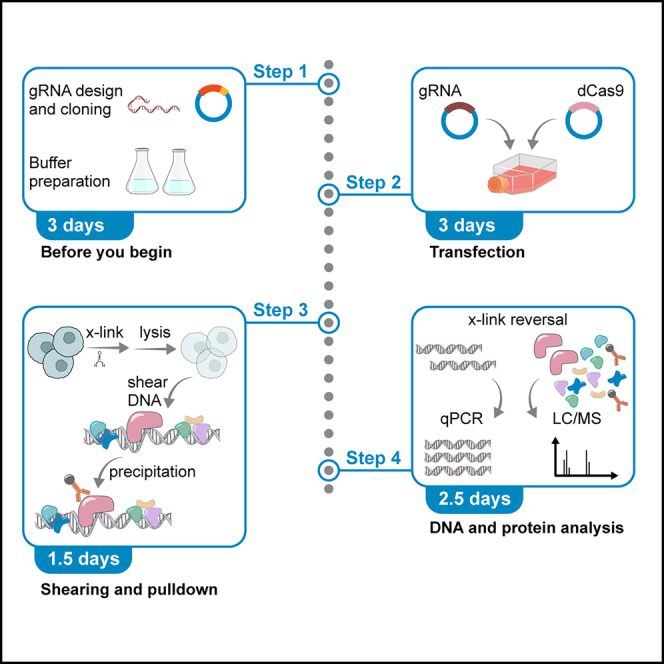

## Before you begin

The analysis of the transcription factors responsible for the expression of a specific gene remains technically challenging as they are only present in low abundance at the site of interest.^2^^,^^3^^,^^4^ Recently we identified potential transcription factors associated with the promoter of the NK cell activating ligand MICA^1^ based on a previously described technique referred to as *in situ* capture of chromatin utilizing an inactive Cas9 (dCas9).^5^ In combination with a guide RNA (gRNA) targeting the promoter region of *MICA*, we applied this method to retain dCas9 at the site of interest, followed by Cas9 antibody-mediated pull-down and subsequent analysis of bound proteins and DNA by mass spectrometry and PCR, respectively.

### Digest vector constructs

**Timing: 4 h**

1. Design gRNA oligonucleotides to target the locus of interest (here: *MICA* promoter).
  ***Note:*** For the oligo design, we used the Zhang Lab design page which was recently shut down but there are several links to guide design tools (e.g. https://zlab.squarespace.com/guide-design-resources).
  a. After designing the oligos, add CACCG to the 5′ end and remove the PAM sequence at the 3′ end of the forward oligo.
  b. Add AAAC to the 5′ end of the reverse oligo.
    ***Note:*** Design the gRNAs to target the region within 50 to 200 nucleotides upstream of the transcription start site.
2. Digest and purify the plasmid.
  a. Mix the digestion reaction using 2 μg gRNA-GFP-T1 plasmid, 2 μL 10× Buffer G, 1 μL *Bbs*I, and add nuclease-free water up to 20 μL.
  b. Incubate the digestion reaction for 2 h at 37°C.
  c. Inactivate the digestion reaction by heating at 65°C for 20 min.
  d. Purify the digested plasmid by using the Thermo Scientific Gene JET PCR Purification Kit (https://www.thermofisher.com/document-connect/document-connect.html?url=https://assets.thermofisher.com/TFS-Assets%2FLSG%2Fmanuals%2FMAN0012662_GeneJET_PCR_Purification_UG.pdf).
  e. Measure the plasmid concentration in a spectrophotometer.

### Preparation of gRNA oligonucleotide pairs

**Timing: 1.5 h**

3. Phosphorylate and anneal the corresponding oligonucleotide pairs.
  a. Prepare the master mix using 1 μL forward oligo, 1 μL reverse oligo [100 μM stock solutions], 1 μL 10× Ligation Buffer, 0.5 μL T4 PNK, and add up to 10 μL with nuclease-free water.
  b. Incubate at 37°C for 30 min.
  c. Inactivate at 95°C for 5 min, then cool to 25°C.
  d. Dilute the annealed oligos (1:250) in nuclease-free water.

### Vector and gRNA ligation

**Timing: 1.5 h**

4. Ligate the plasmid and oligo pairs.
  a. Pipette 50 ng of the digested vector, 2 μL of the annealed and diluted oligos, 2 μL 10× DNA Ligase Buffer, 1 μL T4 Ligase, and nuclease-free water to 20 μL in a 1.5 mL reaction tube.
  b. Incubate the ligation reaction for 1 h at 20°C–22°C.

### Transformation

**Timing: 2.5 days**

5. Transform the plasmid into competent cells.
  a. Thaw *E.coli* XL1-blue competent bacteria on ice for about 20 min.
  b. Add 8 μL of the ligation mixture to 100 μL of the bacteria in a 1.5 mL reaction tube.
  c. Gently mix by hand and place back on ice for 30 min.
  d. Heat shock the competent cells with the ligation mixture by exposing them to 42°C for 90 s.
  e. Cool the mixture on ice for 2 min.
  f. Add 500 μL of antibiotic-free LB medium to the mixture.
  g. Incubate for 2 h at 37°C with shaking at 700 rpm.
  h. Centrifuge the mixture at 300 × *g* for 2 min at 20°C–22°C.
  i. Discard 500 μL of the supernatant.
  j. Gently resuspend the pellet in the remaining medium.
  k. Inoculate an LB agar plate containing Kanamycin (50 μg/mL).
  l. Incubate the plate overnight at 37°C for 16 h.
  m. Store the plate at 4°C before you proceed to the next step.
  n. Pick individual colonies and inoculate in 4 mL LB medium containing Kanamycin (50 μg/mL).
  o. Incubate overnight at 37°C on a shaker for 16 h.
  p. Extract DNA by using the GeneJET Plasmid Miniprep Kit according to the manufacturer’s instructions (https://www.thermofisher.com/document-connect/document-connect.html?url=https://assets.thermofisher.com/TFS-Assets%2FLSG%2Fmanuals%2FMAN0013117_GeneJET_Plasmid_Miniprep_UG.pdf).
  q. Send the purified plasmid to a provider for sequencing services (here: LGC Genomics, Berlin, Germany) using T7 forward primer 5′-TAATACGACTCACTATAGGG-3’.

### Blocking of Sepharose A and G

**Timing: 1 day**

6. Block Sepharose beads A and G.
  a. Mix 5 mL of protein A agarose and 5 mL of protein G agarose in a 50 mL falcon containing 15 mL Lysis Buffer II with freshly added 1× protease inhibitor cocktail for washing.
  b. Centrifuge for 5 min at 1,200 × *g* at 4°C and discard the supernatant.
  c. Repeat this step one more time.
  d. Resuspend the washed beads in 15 mL Lysis Buffer II with freshly added 1× protease inhibitor cocktail containing 1 g/L BSA and 0.4 g/L sonicated salmon sperm DNA.
  e. Incubate the beads for 16 h at 4°C on a roller.
  f. Centrifuge the blocked beads and discard a part of the supernatant (buffer) to have a 50% slurry, meaning if the volume of the beads after centrifugation is 7 mL, leave 7 mL of the buffer and discard the rest.
  g. Aliquot the blocked beads into a 5 mL reaction tube.
  h. Store the beads at 4°C for up to a year.

## Key resources table

Companies are indicated, however, reagents that are available from other sources can also be used, provided they are free of DNA contamination and recommended for cell culture.

**Table:** 

REAGENT or RESOURCE	SOURCE	IDENTIFIER
**Antibodies**		

Mouse anti-Cas9 monoclonal antibody (10 μg/100 μL beads; 4 μg/300 μL chromatin IP control; and 100–120 μg/20 mL soluble chromatin)	Sigma-Aldrich	Cat# SAB4200701; clone 7A9
Rabbit IgG antibody (10 μg/100 μL beads; 4 μg/300 μL chromatin IP control; and 100–120 μg/20 mL soluble chromatin)	Sigma-Aldrich	Cat# i5006-10MG

**Bacterial strain**		

*E. coli* XL1-Blue competent cells	Agilent Technologies	Cat# AGLS200249

**Chemicals, peptides, and recombinant proteins**		

DPBS	Thermo Fisher Scientific	Cat# 12037539
Protein A Sepharose	GE Healthcare	Cat# 17-1279-01
Protein G Sepharose	GE Healthcare	Cat# 17-0618-05
DMEM, high glucose, pyruvate	Thermo Fisher Scientific	Cat# 41966052
Fetal bovine serum (FBS)	Thermo Fisher Scientific	Cat# 10437-028
Trypsin-EDTA solution	Sigma-Aldrich	Cat# T4174-100ML
Opti-MEM I reduced serum media	Thermo Fisher Scientific	Cat# 31985062
Lipofectamine 2000	Thermo Fisher Scientific	Cat# 11668027
Penicillin/Streptomycin (Pen/Strep), 100×	Capricorn Scientific	Cat# PS-B
Bovine serum albumin (BSA) fraction V, NZ-Origin	Carl Roth	Cat #8076.2
Ampicillin, ready-made solution, 100 mg/mL, 0.2 μm filtered	Sigma-Aldrich	Cat# A5354-10ML
Kanamycin	Sigma-Aldrich	Cat# K1377
Sonicated salmon sperm DNA (10 mg/mL stock)	Thermo Fisher Scientific	Cat# 15632011
Agarose	Carl Roth	Cat# 3810.3
RNase A	Thermo Fisher Scientific	Cat# EN0531
Protease inhibitor cocktail	Sigma-Aldrich	Cat# P8340-1ML
Yeast extract	Carl Roth	Cat# 2363.2
Peptone	Carl Roth	Cat# 8986.2
Nuclease-free water	Carl Roth	Cat# T143.6
Formaldehyde 37% or formaldehyde (methanol free), 10% UltraPure EM grade	Carl Roth; Polysciences, Inc.	Cat# 7398.4; Cat#04018-1
Glycine	Carl Roth	Cat# 3908.2
Tris	Carl Roth	Cat# AE15.2
Tris hydrochloride	Carl Roth	Cat# 9090.3
Sodium chloride (NaCl)	Carl Roth	Cat# 3957.1
PIPES	Carl Roth	Cat# 9156.2
Potassium chloride (KCl)	Carl Roth	Cat# 6781.1
Nonidet P40 (NP40)	PanReac AppliChem	Cat# A1694,0250
Sodium deoxycholate	Carl Roth	Cat# D6750-25G
Sodium dodecyl sulfate (SDS)	Carl Roth	Cat# CN30.3
Ethylenediamine tetraacetic acid disodium salt dihydrate (EDTA)	Carl Roth	Cat# 8043.2
Triton X-100	Carl Roth	Cat# 3051.2
Sodium hydrogen carbonate (NaHCO_3_)	Carl Roth	Cat# 6885.1
Lithium chloride (LiCl)	Carl Roth	Cat# P007.1
Isopropanol	Carl Roth	Cat# 6752.4
Ethanol	Carl Roth	Cat# P075.4
Proteinase K	Thermo Fisher Scientific	Cat# EO0491
Methanol	Carl Roth	Cat# 4627.5
Buffer G	Thermo Fisher Scientific	Cat# BG5
*Bbs*I	Thermo Fisher Scientific	Cat# ER1011
T4 DNA ligation buffer	Thermo Fisher Scientific	Cat# B69
T4 DNA ligase	Thermo Fisher Scientific	Cat# EL0011
T4 polynucleotide kinase (PNK)	Thermo Fisher Scientific	Cat# EK0031
Absolute qPCR SYBR Green Mix	Thermo Fisher Scientific	Cat# AB1158B
Mineral oil	Carl Roth	Cat# HP50.1
GelRed nucleic acid gel stain	Biotium	Cat# B-41003

**Experimental model: Cell line**		

HEK293	Leibniz Institute, DSMZ-German Collection of Microorganisms and Cell Cultures GmbH	Cat# ACC 305

**Oligonucleotides**		

T7 fw	TAATACGACTCACTATAGGG	
gRNA MICA_1 fw gRNA MICA_1 rev	CACCGTTAATGGGGCGGCCGGCGAA AAACTTCGCCGGCCGCCCCATTAAC	
gRNA MICA_2 fw gRNA MICA_2 rev	CACCGTAATGGGGCGGCCGGCGAAA AAACTTTCGCCGGCCGCCCCATTAC	
gRNA MICA_4 fw gRNA MICA_4 rev	CACCGTTTCATTGGATGAGCGGTCG AAACCGACCGCTCATCCAATGAAAC	
gRNA MICA_6 fw gRNA MICA_6 rev	CACCGTTCATTGGATGAGCGGTCGG AAACCCGACCGCTCATCCAATGAAC	
gRNA MICA_8 fw gRNA MICA_8 rev	CACCGCCAGAAAATGGGGAGCACGC AAACGCGTGCTCCCCATTTTCTGGC	
gRNA Negative Control fw gRNA Negative Control rev	CACCTTCAGCTCGATGCGGTTCAC AAACGTGAACCGCATCGAGCTGAA	
ChIP-qPCR MICA_fw ChIP-qPCR MICA_rev	CGTGCTTATGAAGTTGGA AGACCTGGGGAGATTTAG	
RT-qPCR MICA_fw RT-qPCR MICA_rev	CTGCAGGAACTACGGCGATATCT CCCTCTGAGGCCTCGCTG	

**Recombinant DNA**		

gRNA-GFP-T1	Addgene	Cat# 41819
pEGFP-C1	Clontech	discontinued
pEF1a-FB-dCas9puro	Addgene	Cat# 100547
SP-dCas9-VPR	Addgene	Cat # 63798

**Critical commercial assays**		

QIAquick PCR purification kit	QIAGEN	Cat# 28106
GeneJET PCR Purification Kit	Thermo Fisher Scientific	Cat# 10400450
GeneJET Plasmid Miniprep Kit	Thermo Fisher Scientific	Cat# K0502
DNA Cleanup Buffers Buffer PE	QIAGEN	Cat# 19065
Buffer EB	QIAGEN	Cat# 19086

**Other**		

EpiShear cooled sonication platform	Active Motif	Cat# 53077
QIAvac 24 Plus	QIAGEN	Cat# 19413
qPCR system	Agilent Technologies	Cat# Mx3000P

## Materials and equipment

**Table:** Buffers and Solutions

LB medium		
Reagent	Final concentration	Amount
Peptone	1% (w/v)	10 g
NaCl	1% (w/v)	10 g
Yeast extract	0.5% (w/v)	5 g
ddH_2_O	N/A	Up to 1 L
**Total**	**N/A**	**1 L**

Note on storage conditions: 4°C, 2 weeks

**Table:** 

LB medium + Kanamycin		
Reagent	Final concentration	Amount
Peptone	1% (w/v)	5 g
NaCl	1% (w/v)	5 g
Yeast extract	0.5% (w/v)	2.5 g
Kanamycin	50 μg/mL	25 mg
ddH_2_O	N/A	Up to 500 mL
**Total**	**N/A**	**500 mL**

Note on storage conditions: 4°C, 2 weeks

**Table:** 

LB agar plate + Kanamycin		
Reagent	Final concentration	Amount
Peptone	1% (w/v)	10 g
NaCl	1% (w/v)	10 g
Yeast extract	0.5% (w/v)	5 g
Agar	1.5% (w/v)	15 g
Kanamycin	50 μg/mL	50 mg
ddH_2_O	N/A	Up to 1 L
**Total**	**N/A**	**1** **L**

Note on storage conditions: 4°C, 2 weeks

**Table:** 

Lysis buffer I		
Reagent	Final concentration	Amount
PIPES (500 mM)	5 mM pH 8.0	1 mL
KCl (1 M)	85 mM	8.5 mL
NP40	0.5% (v/v)	0.5 mL
Protease inhibitor cocktail (Freshly added)	0.1% (v/v)	0.1 mL
ddH_2_O	N/A	Up to 100 mL
**Total**	**N/A**	**100 mL**

Note on storage conditions: without protease inhibitor: 4 °C, 1 year or −20°C, 3 years

**Table:** 

Lysis buffer II		
Reagent	Final concentration	Amount
Tris-HCl (1 M)	10 mM pH 7.5	0.25 mL
NaCl (3 M)	150 mM	1.25 mL
NP40	1% (v/v)	0.25 mL
Sodium deoxycholate	1% (w/v)	0.25 g
SDS	0.1% (w/v)	5 mg
EDTA (500 mM)	1 mM	0.05 mL
Protease inhibitor cocktail (Freshly added)	0.1% (v/v)	0.025 mL
BSA stock (Freshly added)	1 g/L	1.25 mL
Sonicated salmon sperm DNA (10 mg/mL stock, freshly added)	0.4 g/L	1 mL
ddH_2_O	N/A	Up to 25 mL
**Total**	**N/A**	**25 mL**

Note on storage conditions: without protease inhibitor, BSA, and salmon sperm DNA: 4 °C, 1 year or −20°C, 3 years

**Table:** 

BSA stock		
Reagent	Final concentration	Amount
UltraPureTM BSA (Store −20°C)	20 g/L	0.4 g
EB Buffer, QIAGEN	N/A	Up to 10 mL
**Total**	**N/A**	**10 mL**

Note on storage conditions: 4°C, 2 weeks

**Table:** 

Wash buffer I		
Reagent	Final concentration	Amount
Tris (1 M)	20 mM pH 8.1	10 mL
NaCl (3 M)	150 mM	25 mL
EDTA (500 mM)	2 mM	2 mL
SDS	0.1% (w/v)	0.5 g
Triton X-100	1% (v/v)	5 mL
ddH_2_O	N/A	Up to 500 mL
**Total**	**N/A**	**500 mL**

Note on storage conditions: 4 °C, 3 years

**Table:** 

Wash buffer II		
Reagent	Final concentration	Amount
Tris (1 M)	20 mM pH 8.1	10 mL
NaCl (3 M)	500 mM	83.33 mL
EDTA (500 mM)	2 mM	2 mL
SDS	0.1% (w/v)	0.5 g
Triton X-100	1% (v/v)	5 mL
ddH_2_O	N/A	Up to 500 mL
**Total**	**N/A**	**500 mL**

Note on storage conditions: 4 °C, 3 years

**Table:** 

Wash buffer III		
Reagent	Final concentration	Amount
Tris (1 M)	10 mM pH 8.1	1 mL
LiCl (1 M)	250 mM	25 mL
NP40	1% (v/v)	1 mL
Sodium deoxycholate	1% (w/v)	1 g
EDTA (500 mM)	1 mM	0.2 mL
ddH_2_O	N/A	Up to 100 mL
**Total**	**N/A**	**100 mL**

Note on storage conditions: 4 °C, 3 years

**Table:** 

Elution buffer (400 μL per sample)		
Reagent	Final concentration	Amount
SDS	1% (w/v)	50 μL
NaHCO_3_ (1 M)	100 mM	500 μL
ddH_2_O	N/A	Up to 5 mL
**Total**	**N/A**	**5 mL**

Note on storage conditions: individual components: 20°C–22°C, 1 month; otherwise, prepare fresh

**Table:** 

Reversion mix buffer (42 μL per sample)		
Reagent	Final concentration	Amount
NaCl (5 M)	1.67 M	16 μL
Tris (1 M)	1.67 M	16 μL
EDTA (500 mM)	167 mM	8 μL
RNase A (10 g/L)	10 μg	1 μL
Proteinase K (20 g/L)	20 μg	1 μL
**Total**	**N/A**	**42 μL**

Note on storage conditions: without RNase A and Proteinase K: 20°C–22°C, 1 year; otherwise, prepare fresh.

## Step-by-step method details

### Cell culture transfection for the ChIP assay

**Timing: 3 days**

This step describes the transfection of HEK293 cells with dCas9 and gRNA vectors (designed in the “before you begin” section) that target the locus of interest (here: *MICA* promoter).

***Note:*** Human embryonic kidney cells (HEK293) are used in the following steps.

***Note:*** This protocol provides the volumes for a single 15 cm culture dish for each condition. 10 × 15 cm dishes were used per condition to obtain sufficient cell numbers for mass spectrometry analysis.

1. Transfect cells with dCas9 and gRNA vectors.
  a. Thaw the cells and culture them in DMEM medium containing 10% FBS and 1% penicillin/streptomycin at 37°C with 5% CO_2_.
  b. Passage the cells twice to three times per week and use them when the passage number is between two and five.
  c. Seed 5–6 × 10^6^ cells in a 15 cm cell culture dish containing 20 mL DMEM medium with 10% FBS and 1% penicillin/streptomycin 20 h before transfection.
  d. 3 h prior to transfection, replace the medium with penicillin/streptomycin-free DMEM with 10% FBS.
  e. For transfection, dilute 40 μL Lipofectamine 2000 DNA Transfection Reagent in 750 μL serum-free Opti-MEM in a 2 mL reaction tube A.
  f. In a 2 mL reaction tube B add 750 μL Opti-MEM and 16 μg DNA (8 μg pEF1a-FB-dCas9-puro + 8 μg gRNA-MICA-T1 vectors).
  g. Mix both tubes by vortexing.
  h. Incubate the tubes for 5 min at 20°C–22°C.
  i. Add the content of tube A to tube B and mix by pipetting.
  j. Incubate for 20 min at 20°C–22°C.
  k. Add the transfection mixture dropwise to the 15 cm cell culture dish containing HEK293 cells in a circular motion.
  l. Incubate the dish at 37°C with 5% CO_2_ for approximately 17 h.
  m. The next day, replace the transfection medium with DMEM medium containing 10% FBS and 1% penicillin/streptomycin.
  n. Evaluate transfection efficiency using pEGFP-C1 plasmid as a control and visualize by fluorescence microscopy.
  o. 48 h post-transfection, subject cells to engineered DNA binding molecule-mediated chromatin immunoprecipitation (enChIP) assay.

### Precipitation of chromatin via dCas9

**Timing: 1.5 days**

This section describes the dCas9-mediated precipitation of the chromatin followed by shearing and subsequent incubation with beads.

2. Prepare native chromatin.
  a. Fix cells by adding 2 mL formaldehyde (10% stock) to 18 mL of medium for a final concentration of 1%.
  b. Gently swirl the plate.
    ***Note:*** The color of the medium will change from pink to yellow for a few minutes.
  c. Incubate for 10 min at 20°C–22°C under a chemical fume hood.
  d. Quench the formaldehyde by adding 1 M glycine to a final concentration of 125 mM.
  e. Mix by gentle swirling.
  f. Incubate for 5 min at 20°C–22°C.
  g. Discard the supernatant and wash the cells twice with cold DPBS.
  h. Scrap the cells with a rubber policeman, harvest with a 1000 μL pipette tip, and transfer to a 15 mL falcon.
  i. Centrifuge the cells at 1200 × *g* for 5 min at 4°C.
  j. Discard the supernatant.
    ***Note:*** In this step, the cell pellet can be stored at – 80°C for up to a year.
  k. Gently but thoroughly resuspend the pellet in 2 mL Lysis Buffer I (add fresh protease inhibitor cocktail 1:1,000) using a 1000 μL pipette tip to obtain a homogenous suspension.
  l. Incubate for 20 min on ice.
    ***Note:*** This step is to disrupt the cellular membrane and remove the cytoplasmic components.
  m. Centrifuge at 1,200 × *g* for 5 min at 4°C.
  n. Discard the supernatant.
  o. Gently but thoroughly resuspend the nuclei in 2 mL Lysis Buffer II (add fresh protease inhibitor cocktail 1:1,000).
  p. Incubate on ice for 10 min.
  q. Ensure that the suspension is homogeneous and carefully transfer 1 mL each into two new 15 mL falcons with a 1,000 μL pipette tip.
    **CRITICAL:** Avoid the formation of air bubbles. It is critical for the chromatin sonication step to prevent protein denaturation at the surface and loss of chromatin in the bubbles, which may affect the DNA/protein complex required for the precipitation step.
3. Shear the chromatin.
  **CRITICAL:** For the sonication step, it is essential to keep the pellet cool, which can be achieved by using a sonication system, here the Benchtop Tube Cooler, Active Motif, no. 53077.
  ***Note:*** Always store the 15 mL Benchtop Tube Cooler at −20°C and remove it before use. It can be stored at −80°C but for no longer than 30 min.
  ***Note:*** To avoid freezing the sample, do not place the sample in the cooler until you are ready to sonicate it directly.
  ***Note:*** The setting of the sonication step must be defined for each cell line and sonication device.
  a. Set the sonication device according to the cell line (here: HEK293 cell line) as follows: in total 52 pulses of 1 s pulse with 4 s pause at 20% amplitude.
  b. Fill the 15 mL Benchtop Tube Cooler with 1 mL NaCl (5 M) using a 1000 μL pipette tip.
  c. Place the falcon in the cooler and directly into the sonicator.
    **CRITICAL:** The 15 mL falcon should be positioned in the center of the cooler without touching the edge.
    **CRITICAL:** Try to preserve the epitopes by sonicating the samples as weakly and briefly as possible. Otherwise, indirectly bound proteins can easily be lost during the procedure.
    **CRITICAL:** To avoid foaming of the sample, immerse the tip of the sonicator at 60–80% of the maximum depth. In the case of foaming, the epitope will be affected by oxidation and the shear efficiency will be drastically reduced.
  d. Transfer the sheared chromatin to a new 1.5 mL reaction tube.
  e. Centrifuge at 13,000 × *g* for 15 min at 4°C.
    ***Note:*** This is the soluble chromatin fraction.
  f. Collect 2 mL of soluble chromatin per 15 cm dish.
    ***Note:*** In this study, a total of 20 mL per condition (10 × 15 cm dishes per condition) was used.
4. Determine shearing efficiency.
  a. Pool the soluble chromatin from twenty 1.5 mL reaction tubes into two 15 mL falcons for the next steps.
  b. To determine the chromatin shear efficiency, transfer approximately 10 μL to a new reaction tube.
  c. Add 20 μg Proteinase K, 20 μg RNase A, and 100 μL mineral oil.
  d. Mix well by vortexing.
  e. Incubate for 16 h at 65°C.
  f. Mix 10 μL of the sample with loading buffer and load onto a 1% agarose gel containing GelRed.
  g. Run the gel at a maximum voltage of 80 V and check the size of the DNA fragments.

***Note:*** DNA fragments should be between 500 bp and 700 bp.

**CRITICAL:** The appearance of the pellet can provide information about the sonication efficacy; if the pellet is large and white, it means that the sonication was insufficient. If the pellet is small and transparent with dark speckles, the number of pulses or the amplitude, or both, must be reduced to conserve the epitope.

5. Prepare the blocked beads for incubation with chromatin.
  a. Use a 50% slurry of blocked beads and incubate with a non-specific antibody such as rabbit IgG antibody.
  b. Add 10 μg IgG per 100 μL of beads.
  c. Incubate at 4°C for 30 min on a roller.
    **CRITICAL:** Prepare enough blocked beads for all samples, 100 μL of the 50% slurry beads blocked with IgG for the 1 mL of the soluble chromatin. In total 2 mL of the beads for each 20 mL of the soluble chromatin.
  d. Centrifuge the blocked beads at 300 × *g* for 1 min at 4°C.
  e. Carefully aspirate the supernatant.
  f. Wash the blocked beads twice with Lysis Buffer II (with protease inhibitor cocktail 1:1,000).
  g. Centrifuge for 1 min at 300 × *g* at 4°C.
  h. Discard the supernatant.
  i. Add sufficient buffer to achieve 50% slurry of the beads.
  j. Repeat steps f to i.
    **CRITICAL:** Gently handle the beads, taking care not to disrupt them during centrifugation and aspiration of the supernatant; you can use a vacuum system (QIAvac 24 Plus, QIAGEN) connected to a needle to aspirate the supernatant.
  k. For chromatin pre-clearing, add 2 mL of the 50% slurry of the beads (blocked with IgG antibody) to 20 mL of the soluble chromatin.
  l. Incubate for 45 min at 4°C on a roller.
  m. Centrifuge at 1,200 × *g* for 5 min at 4°C.
  n. Transfer the pre-cleared chromatin to a new falcon tube.
  o. Combine the two 15 mL falcon tubes of the soluble chromatin in one 50 mL falcon tube.

### Elution of DNA and proteins plus analysis

**Timing: 2.5 days**

In the following section, the precipitated DNA and proteins are isolated and prepared for PCR and mass spectrometric analysis, respectively.

6. Immunoprecipitate the chromatin (carry this step out in parallel with step 12).
  a. At this time, collect an aliquot of soluble chromatin equal to 1% of the volume used for immunoprecipitation (IP) and store it at 4°C; this is referred to as the input sample.
    ***Note:*** In this protocol, 3 μL of soluble chromatin is the required volume.
  b. Add 4 μg of IgG antibody to 300 μL of soluble chromatin (for the IP control).
  c. Add 100–120 μg of Cas9 antibody to the remaining volume of soluble chromatin (approximately 20 mL).
  d. Incubate the samples for 16 h at 4°C on a roller.
    ***Note:*** Store the input samples at 4°C for later use.
  e. Transfer 300 μL of the Cas9-IP into a new reaction tube to proceed with the qPCR assay and confirm precipitation of the target locus. The rest of the Cas9-IP sample is for mass spectrometry analysis.
  f. For each IP, add 50 μL of the A + G blocked beads slurry (from blocking of Sepharose beads A and G) and rotate for 1 h at 4°C.
    **CRITICAL:** Before using the A + G blocked beads slurry, gently mix to obtain a homogeneous suspension. Try to mix gently by inverting the reaction tube before pipetting each sample.
    ***Note:*** To remove the 50 μL of bead suspension, use the 200 μL pipette tips and cut off the end with clean scissors. This will make it easier to pipette the 50% bead suspension.
7. Perform a qPCR to detect the target locus (here *MICA*) using the following 3 samples:
  a. input sample,
  b. chromatin precipitated with Cas9 antibody (Cas9-IP), and
  c. chromatin precipitated with control antibody (IgG-IP).

**Table:** PCR reaction master mix

Reagent	Amount
DNA	1 μL
SYBRGreen Mix	5 μL
10 μM ChIP-qPCR MICA_fw	0.25 μL
10 μM ChIP-qPCR MICA_rev	0.25 μL
H_2_O	3.5 μL

**Table:** PCR cycling conditions

Steps	Temperature	Time	
Initial Denaturation	95°C	15 min	1
Denaturation	95°C	20 s	40 cycles
Annealing	60°C	15 s	
Extension	72°C	60 s	
Final extension	72°C	5 min	1
Hold	4°C	∞

8. Wash the beads.
  a. Centrifuge the IP samples at 300 × *g* for 1 min at 4°C.
  b. Discard the supernatant.
  c. Add 1 mL of cold Wash Buffer I to each IP sample and carefully invert the reaction tube.
  d. Centrifuge at 300 × *g* for 1 min at 4°C.
  e. Discard the supernatant.
  f. Wash the IP sample with 1 mL of cold Wash Buffer II and mix gently by inversion.
  g. Centrifuge at 300 × *g* for 1 min at 4°C.
  h. Discard the supernatant.
  i. Wash the IP sample with 1 mL cold Wash Buffer III.
  j. Centrifuge at 300 × *g* for 1 min at 4°C.
  k. Discard the supernatant.
  l. Repeat steps i to k.
  m. Add 1 mL elution buffer (EB buffer, QIAGEN) to each IP sample and mix gently.
  n. Centrifuge at 300 × *g* for 1 min at 20°C–22°C.
  o. Discard the supernatant.
  p. Repeat steps m to o.

**CRITICAL:** Keep the samples on ice for steps a to m.

**CRITICAL:** Handle the beads with care and aspirate the supernatant without disturbing the beads.

***Note:*** For bead washing steps, you can use a vacuum system (QIAvac 24 Plus, QIAGEN) that you connect to a special needle to aspirate the supernatant.

9. Elute immunoprecipitates from the beads.
  ***Note:*** For this step, prepare 400 μL of freshly prepared elution buffer for each sample (also include the input samples in this step).
  a. Incubate the beads for each sample with 200 μL of elution buffer for 15 min at 20°C–22°C with vigorous shaking (1,200 rpm).
  b. Centrifuge the samples at high speed (17,000 × *g*) for 2 min at 20°C–22°C.
  c. Carefully transfer the supernatant to a new reaction tube.
  d. Repeat steps a and b. Then combine the supernatants.
  e. Add 400 μL elution buffer to each of the input samples.
    **CRITICAL:** Carefully transfer the supernatant into a new 1.5 mL reaction tube without any beads. Use the 200 μL pipette tip to transfer the supernatant.
  f. Prepare fresh Reversion Mix Buffer and add 42 μL to each sample.
  g. Mix well by vortexing.
  h. Incubate for 16 h at 65°C.
    ***Note:*** Following 16 h incubation, you may store the samples at 4°C and purify the DNA up to two days later.
10. Purify the DNA using the QIAGEN PCR Purification Kit.
  a. Mix each sample with 2.2 mL of Binding Buffer PB (in a 5 mL reaction tube) to obtain a clear suspension.
  b. Apply the sample to the provided QIAquick Spin Column.
  c. Centrifuge at 17,000 × *g* for 1 min at 20°C–22°C and discard the flow through.
  d. Add 1 mL of PE Buffer to the wash.
  e. Centrifuge at 17,000 × *g* for 1 min at 20°C–22°C.
  f. Transfer the filter to a new reaction tube.
  g. Add 30 μL of elution buffer per sample and incubate for 2 min at 20°C–22°C.
  h. Centrifuge at high speed for 2 min at 20°C–22°C.

***Note:*** Samples are ready for qPCR analysis using primers for the target region (1 μL is sufficient for each sample). Perform the qPCR in technical triplicates.

11. Perform a qPCR to quantify the target DNA region.
  **Table:** PCR reaction master mix

Reagent	Amount
DNA	1 μL
SYBRGreen Mix	5 μL
10 μM ChIP-qPCR MICA_fw	0.25 μL
10 μM ChIP-qPCR MICA_rev	0.25 μL
H_2_O	3.5 μL
  **Table:** PCR cycling conditions

Steps	Temperature	Time	
Initial Denaturation	95°C	15 min	1
Denaturation	95°C	15 s	40 cycles
Annealing	60°C	20 s	
Extension	72°C	15 s	
Denaturation	95°C	60 s	1
Starting temperature	72°C	30 s	1
Melting curve	72°C–95°C	10 s	0.5°C per cycle

  a. Calculate the enrichment of the target DNA sequence after precipitation with the selected antibody as a percentage of the input:
    $$\text{Input}\;\%=2^{-(\text{CT}\;\text{sample}\;-\;\text{CT}\;\text{input})}$$
    ***Note:*** The input sample presents 1% of soluble chromatin in this protocol.
12. Prepare Cas9-IP samples for mass spectrometry (carry out this step in parallel with step 6).
  a. For precipitation, add 1.5 mL of A + G blocked beads to the Cas9 IP sample.
  b. Incubate for 1 h at 4°C on a roller.
  c. Centrifuge at 1,200 × *g* for 2 min at 4°C and aspirate the supernatant.
  d. Add 20 mL Wash Buffer I and gently invert the falcon.
  e. Centrifuge at 1,200 × *g* for 2 min at 4°C and aspirate the supernatant.
  f. Wash the sample with 20 mL Wash Buffer II and mix gently.
  g. Centrifuge at 1,200 × *g* for 2 min at 4°C and aspirate the supernatant.
  h. Add 20 mL Wash Buffer III and mix gently.
  i. Centrifuge at 1,200 × *g* for 2 min at 4°C and aspirate the supernatant.
  j. Repeat steps h to i.
    **CRITICAL:** Keep samples on ice during the previous washing steps and handle beads carefully. Do not disturb the beads. You can use a vacuum system (QIAvac 24 Plus, QIAGEN) that you connect to a special needle to aspirate the supernatant.
  k. Wash the sample with 20 mL EB buffer (QIAGEN) twice.
  l. Centrifuge at 1,200 × *g* for 2 min at 4°C and aspirate the supernatant.
  m. Store beads at −80°C in 200 μL EB buffer containing 1× protease inhibitor cocktail until subjected to off-bead digestion.
  n. For off-bead digestion use 6 M Urea / 2 M Thiourea with 10 mM DTT and high pH reversed-phase separation followed by LC-MS2 analysis using label-free quantitation.

## Expected outcomes

The procedure described above allowed the successful pull-down of proteins at the chromatin locus of interest, i.e. the *MICA* promoter region. However, the identification of transcription factors or their networks remains difficult. The enChIP pull-down and subsequent mass spectrometric analysis of the associated factors yielded high amounts of background proteins including histones, polymerases, and DNA repair factors in addition to low-abundance transcription factors (3), which are detected in both, the negative control (GFP) and the MICA samples. A strategy to filter promising candidates is to i) focus on already established binding factors (provided such information is available), which are enriched in the MICA samples compared to the GFP samples and ii) to look for factors with corresponding binding motifs for the locus. Potential candidates should then further be validated in follow-up experiments such as luciferase reporter assays. For the success of the approach, the following aspects are critical: i) the design of several gRNAs targeting the locus of interest. These gRNAs are validated by a CRISPR-mediated transcriptional activation system followed by qPCR, to analyze whether the gRNAs show the ability to activate the transcription of *MICA* using specific primers (RT-qPCR MICA, see list). ii) The pull-down was performed using a Cas9 antibody, which was more specific and efficient compared to a Streptavidin/Biotin approach in our hands.

## Limitations

The unbiased approach comes along with the precipitation of nuclear and chromatin-associated proteins, which are not involved in locus-specific transcription. Therefore, a negative control, in our case cells transfected with a non-targeting gRNA, should be run in parallel. The “technical noise” can then be subtracted from the data set. Despite using 10 × 15 cm dishes the yield of transcription factors remains low and variation between samples remains high, therefore more than three independent experiments are necessary for reliable detection and statistical analysis.

## Troubleshooting

### Problem 1

The signal of ChIP-qPCR is low. Generally, there are multiple possible reasons for low signal-to-noise ratios, such as the nature or concentration of the antibody or too little starting material. Here, we think that the sonication is a critical step, which can lead to longer DNA fragments (a cause for non-specific qPCR amplification) or shorter DNA fragments (are lost during the purification step).

### Potential solution

300 to 600 base pair fragments of DNA are optimal and the number, duration (pulse/pause cycles), and amplitude of sonification should be optimized (steps 3 and 4).

### Problem 2

Proteins are lost during the procedure, due to sonification-mediated epitope degradation.

### Potential solution

Samples should be sonicated as weakly and briefly as possible and foaming of the sample should be avoided.

### Problem 3

Few proteins are precipitated.

### Potential solution

It is critical to carefully handle the beads and avoid any damage caused by centrifugation, pipetting, or aspiration of the supernatant (steps 5, 6, and 8).

### Problem 4

The qPCR (step 9) fails to detect enrichment of the target locus (here *MICA*).

### Potential solution

Design gRNAs with different target sequences and always use a mix of gRNAs, which targets the locus of interest.

### Problem 5

You are not sure that the designed gRNAs target the promoter region of interest.

### Potential solution

Use the CRISPR activation system: It consists of inactive dCas9 coupled to the transcriptional activator (VPR) vector (SP-dCas9-VPR, Addgene catalog # 63798). Transfect (transiently) the gRNA vector together with the dCas9-VPR vector and then perform RT-qPCR analysis to evaluate whether the transcription of the target gene is activated. Induced transcription of your gene of interest confirms that the designed gRNA is successfully targeting the locus of interest.

## Resource availability

### Lead contact

Further information and requests for resources and reagents should be directed to and will be fulfilled by the lead contact, Elke Pogge von Strandmann (poggevon@staff.uni-marburg.de).

### Technical contact

Technical questions should be directed to and will be fulfilled by the technical contact Vivian Ponath (ponath@staff.uni-marburg.de).

### Materials availability

This study did not generate any new unique results.

### Data and code availability

This study did not generate/analyze datasets or code.

## References

[1] Alkhayer R., Ponath V., Frech M., Adhikary T., Graumann J., Neubauer A., von Strandmann E.P. (2023). KLF4-mediated upregulation of the NKG2D ligand MICA in acute myeloid leukemia: a novel therapeutic target identified by enChIP. Cell Commun. Signal..

[2] Gauchier M., van Mierlo G., Vermeulen M., Déjardin J. (2020). Purification and enrichment of specific chromatin loci. Nat. Methods.

[3] Vermeulen M., Déjardin J. (2020). Locus-specific chromatin isolation. Nat. Rev. Mol. Cell Biol..

[4] Adhikary T., Müller R. (2013). In vivo studies of PPAR-chromatin interactions: chromatin immunoprecipitation for single-locus and genomewide analyses. Methods Mol. Biol..

[5] Liu X., Zhang Y., Chen Y., Li M., Zhou F., Li K., Cao H., Ni M., Liu Y., Gu Z. (2017). In Situ Capture of Chromatin Interactions by Biotinylated dCas9. Cell.

